# On the divide between animate and inanimate

**DOI:** 10.1186/s13322-015-0008-8

**Published:** 2015-02-19

**Authors:** Arto Annila, Erkki Kolehmainen

**Affiliations:** Department of Physics, FI-00014 University of Helsinki, Helsinki, Finland; Institute of Biotechnology, FI-00014 University of Helsinki, Helsinki, Finland; Department of Biosciences, FI-00014 University of Helsinki, Helsinki, Finland; Department of Chemistry, FI-40014 University of Jyväskylä, Jyväskylä, Finland

**Keywords:** Free energy, Life, Non-determinism, The principle of least action, The second law of thermodynamics, Scale invariant

## Abstract

Vitalism was abandoned already for a long time ago, yet the impression that animate beings differ in some fundamental way from inanimate objects continues to thrive. Here, we argue that scale free patterns, found throughout nature, present convincing evidence that this demarcation is only imaginary. Therefore, all systems ought to be regarded alike, i.e., all are consuming free energy in least time. This way evolutionary processes can be understood as a series of changes from one state to another, so that flows of energy themselves naturally select those ways and means, such as species and societies or gadgets and galaxies to consume free energy in the least time in quest of attaining thermodynamic balance in respective surroundings. This holistic worldview, albeit an accurate account of nature, was shelved soon after its advent at the turn of the 18^th^ century, because the general tenet did not meet that time expectations of a deterministic law, but now it is time to reconsider the old universal imperative against observations rather than expectations.

## Background

The recent perspective on *The nature and mathematical basis for material stability in the chemical and biological worlds* by Robert Pascal and Addy Pross elaborates on conceptual conundrums that hinder us from relating animate to inanimate [1]. The authors recap these theoretical problems in the perennial question, how life *could have* emerged from inanimate matter. Moreover, Pascal and Pross are alarmed that biology, as a discipline, has by today grown apart from physical sciences, although chemistry *did* become biology on this planet some 3.5 to 4 billion years ago. Hence, the logical conclusion is that all disciples must have a common conceptual basis.

We have hardly anything to add on this sharp analysis of the status quo. Yet, we wish to emphasize that only by convention we refer to some systems as living while others as nonliving, but nature itself does not make the divide: scale free patterns are ubiquitous [2-10]. Throughout nature are found skewed, nearly log-normal distributions that accumulate along sigmoid curves, and hence appear on log-log scales mostly as straight lines, i.e., comply with power laws [11,12]. These patterns share the same mathematical form, only parameters differ from one system to another.

For example, lengths of genes distribute in the same skew manner as lengths of words. Animal and plant populations, irrespective of a species, spread out on terrestrial and marine environments in the same manner as economic wealth, irrespective of assets, spreads out in diverse societies. Likewise, chemical reactions and economic transactions proceed along sigmoid curves toward stationary cycles such as citric acid cycle in a cell and annual cycles of production. Also a cyclone whirls in a temperature gradient in the same way as a galaxy spirals in the universal density. These logarithmic spirals appear also in many other familiar forms such as shells, cones and inflorescences.

Moreover, ecological succession advances in the same way as technological progress, that is, by punctuating from one innovation to another along a sigmoid curve. Production of goods branches out just as a phylogenic tree of species fans out. So does also an electric discharge disperse in a medium, for instance, lighting in the air. Furthermore, neural activity recorded from cortex follows a power law just as seismic activity recorded from Earth’s mantle. A metabolic network across a cell displays the same power-law degree distribution of intersections as the nodes of a transportation network across a city or the communication network World Wide Web across the Globe as well as the network of galaxies across the Universe. These universal patterns present compelling evidence that there is a natural law that encompasses everything.

### The law of nature

The basic law of nature is no mystery. It says that energy differences of any kind, i.e., free energy of any kind will be consumed in least time when a system of any kind moves from one state to another [12-15]. The opening words of *Principia* address the same relation between forces and motion: *Rational Mechanics will be the science of motions resulting from any forces whatsoever and of the forces required to produce any motions, accurately proposed and demonstrated* [16]. Subsequently Newton produces the renowned equality **F** = d_*t*_**p** between the force **F** and the change in momentum **p**, i.e., the change in the course. No system has any choice but to move along the resultant force, i.e., along the path where free energy is consumed in least time.

Also Carnot recognizes the universality without divide between animate and inanimate [17]: *All substances in nature can be employed for the production of impelling power*. Power *P* equals the consumption of free energy. Since *P* = **F** · **v** = d**p**_*t*_ · **v** = d_*t*_2*K*, no energy in motion with velocity **v**, i.e., kinetic energy 2*K*, has any option but to direct along the steepest gradient on the energy landscape, i.e., along the least-time path.

For example, a brook will vary its path, and this flow of energy will all by itself, i.e., naturally, select the steepest descent to run down the hill slope as soon as possible. Conversely, any rivulet cannot but drain dry when the flow finds a faster way to consume gravitational potential. According to this tenet also an animal population will vary its ways of making living, and the associated flows of energy will themselves naturally select among alternatives, e.g., genetic, epigenetic, behavioral mechanisms, or any other function that facilitates the least-time free energy consumption. Conversely, no species has any freedom but to adapt or perish, when more effective consumers of a common free energy reservoir come around. These courses toward thermodynamic balance with superior surroundings are not unlikely processes, but natural for all systems. It is only a trivial mathematical exercise to show that the least-time imperative, in the form of equation of motion, gives rise to the scale free patterns [12]. Of course, these patterns have been recognized and modeled by various mathematical functions already for a long time, however, the insight that the scale free patterns result from the least-time free energy consumption has been overlooked.

As Pross and Pascal point out, Darwin’s tenet is only a catching narrative without a firm mathematical form. In contrast the universal imperative of least-time free energy consumption as given by Newton’s second law of motion, Maupertuis’ principle of least action or Carnot’s the second law of thermodynamics can be rigorously analyzed [18,19]. These three forms are, in fact, equivalent to each other. Specifically, Newton’s second law of motion can be proven identical to Carnot’s second law of thermodynamics. Recalling that **v** = d_*t*_**x** and 2*K* = **p** · **v** = *TS*, the force **F** = d_*t*_**p** = d_*x*_2*K* = *T*d_*x*_*S* = d_*x*_*Q* is equated with temperature *T* multiplied by the change in entropy d*S* along the piece of path d**x**, which, in turn, is caused by the change in energy d*Q* along d**x**. The mathematical equivalence leaves us without any options but to conclude that evolutionary courses advance along the direction of resultant force. This path of least-time free energy consumption is, in turn, equivalent to the path of maximal rate of entropy increase. However, the mathematical formalism does not imply determinism. On the contrary, evolutionary courses are inherently intractable, because the driving forces are consumed by motions [20]. In other words, the net force keep changing hand in hand with changes in motion, i.e., with evolution.

It is worth emphasizing that the standard way to omit the change in mass d*m*, equal to energy d*E* = d*mc*^2^ dissipated to the vacuum characterized *c*^2^, from the complete form **F** = d_*t*_**p** = *m***a** + **v**d_*t*_*m*, where **a** = d_*t*_**v**, deprives us from understanding any change from one state to another, i.e., evolution. Not even a simple chemical reaction can be understood without dissipation, and hence the textbook thermodynamics and kinetics appear as incongruent. In reality no kinetics runs a reaction, but kinetics is a manifestation the least-time consumption of free energy. Likewise, conceptual conundrums will arise, when entropy is mistaken for a measure of disorder, i.e., incoherence, instead of appreciating it as a sum of both bound and free forms of energy. At the maximum entropy state of a thermodynamic balance all energy is bound, since all free energy has been consumed.

Protein folding, for instance, is obviously not determined by an amino acid sequence alone, but depends also on dissipation to the surroundings whose properties, temperature, pH, ionic strength, chaperons, etc. have a say on the outcome [21]. Therefore, many a biologist rightfully regards the standard deterministic or statistical or probabilistic forms of physics as insufficient to explain life. Conversely, many a physicist shuns the old but accurate dissipative equation of motion, because it cannot be solved. The trouble is not complexity; the trouble is that motion itself affects its driving forces. Hence, evolutionary paths are intractable, but not arbitrary [20]. But isn’t this non-determinism, accompanied with a sense of direction, precisely an outward characteristic of nature? Thus, what cannot be eschew’d must be embrac’d.

## Conclusion

Newton’s, Maupertuis’ and Carnot’s understanding of natural processes across all scales was once as a breath-taking theory as it is today. What the pioneers reasoned complies with reality, and hence with common sense, but not with expectations of a deterministic law. Later, when discrepancy between the looked-for clockwork idealism and reality grew indisputable, imperfect deterministic interpretations of the pioneers’ original prints were not reconsidered, but science went on with disciplinary specialization. As a result, today we find many approximate mathematical models of nature to mimic many a data but to provide only little understanding of underlying causes. Hence, we should return to the exact, albeit intractable evolutionary equation to gain complete comprehension of nature.

Every time in the past when our delusions about uniqueness and particularity have narrowed, our worldview has widened toward entirety. By the same token, we should no longer imagine that animate would be qualitatively distinct from inanimate. Amazing diversity and awesome complexity in mechanistic details, which have accumulated over eons, should no longer distract us from seeing that both simple and sophisticated systems follow the universal principle of least-time free energy consumption.
